# Identification and Re-consent of Existing Cord Blood Donors for Creation of Induced Pluripotent Stem Cell Lines for Potential Clinical Applications

**DOI:** 10.1093/stcltm/szac060

**Published:** 2022-09-08

**Authors:** Keren M Abberton, Tricia L McDonald, Mary Diviney, Rhonda Holdsworth, Stephen Leslie, Martin B Delatycki, Lin Liu, Guy Klamer, Phillip Johnson, Ngaire J Elwood

**Affiliations:** BMDI Cord Blood Bank, Melbourne, Australia; Murdoch Children’s Research Institute, Melbourne, Australia; Department of Surgery, University of Melbourne, Melbourne, Australia; BMDI Cord Blood Bank, Melbourne, Australia; Murdoch Children’s Research Institute, Melbourne, Australia; VTIS at Australian Red Cross Lifeblood, Melbourne, Australia; VTIS at Australian Red Cross Lifeblood, Melbourne, Australia; Schools of Mathematics and Statistics, and BioSciences, Melbourne Integrative Genomics, University of Melbourne, Melbourne, Australia; Murdoch Children’s Research Institute, Melbourne, Australia; Department of Pediatrics, University of Melbourne, Melbourne, Australia; Victorian Clinical Genetics Services, Melbourne, Australia; BMDI Cord Blood Bank, Melbourne, Australia; Murdoch Children’s Research Institute, Melbourne, Australia; Sydney Cord Blood Bank, Sydney Children’s Hospitals Network, Sydney, Australia; Queensland Cord Blood Bank At The Mater, Brisbane, Australia; BMDI Cord Blood Bank, Melbourne, Australia; Murdoch Children’s Research Institute, Melbourne, Australia; Department of Pediatrics, University of Melbourne, Melbourne, Australia

**Keywords:** cord blood and cord tissue banking, ethics, donor re-consent, induced pluripotent stem cell (iPSC), HLA haplotype

## Abstract

We aim to create a bank of clinical grade cord blood-derived induced pluripotent stem cell lines in order to facilitate clinical research leading to the development of new cellular therapies. Here we present a clear pathway toward the creation of such a resource, within a strong quality framework, and with the appropriate regulatory, government and ethics approvals, along with a dynamic follow-up and re-consent process of cord blood donors from the public BMDI Cord Blood Bank. Interrogation of the cord blood bank inventory and next generation sequencing was used to identify and confirm 18 donors with suitable HLA homozygous haplotypes. Regulatory challenges that may affect global acceptance of the cell lines, along with the quality standards required to operate as part of a global network, are being met by working in collaboration with bodies such as the International Stem Cell Banking Initiative (ISCBI) and the Global Alliance for iPSC Therapies (GAiT). Ethics approval was granted by an Institutional Human Research Ethics Committee, and government approval has been obtained to use banked cord blood for this purpose. New issues of whole-genome sequencing and the relevant donor safeguards and protections were considered with input from clinical genetics services, including the rights and information flow to donors, and commercialization aspects. The success of these processes has confirmed feasibility and utility of using banked cord blood to produce clinical-grade iPSC lines for potential cellular therapies.

## Graphical Abstract

**Figure:**
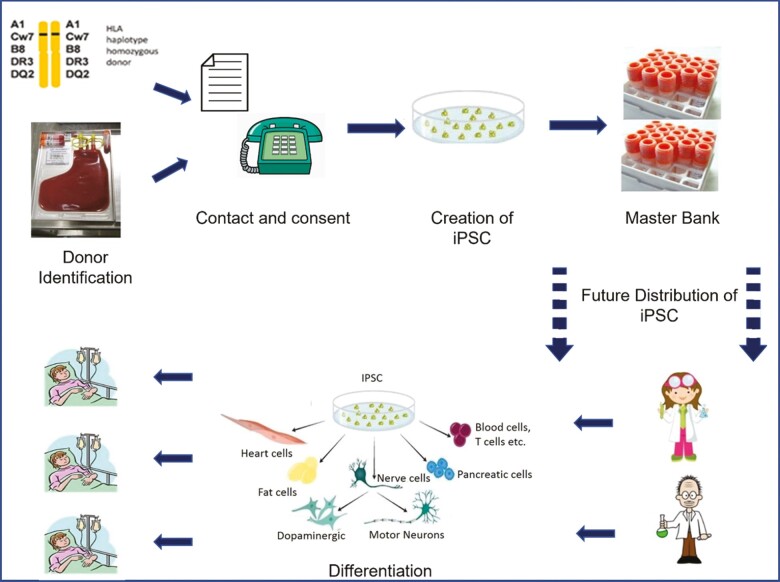

**Significance Statement**

The ethics of using donated cord blood for research is currently an area of great interest, especially relating to induced pluripotent stem cell (iPSC) creation, potential commercial applications, and cellular therapies. We present a clear pathway toward the creation of such a resource with the appropriate regulatory, government, and ethics approvals and donor protections. We have shown donor acceptance of these processes and feasibility and utility of using banked cord blood to produce clinical-grade iPSC. The creation of these iPSC lines will provide an important tool for preclinical research for a range of conditions, leading to the development of new cellular therapies.

## Introduction

Induced pluripotent stem cells (iPSC) are created by reprogramming human somatic cells, such as skin cells or blood cells, into stem cells, using a combination of differentiation factors.^1^ Clinical grade iPSC, compliant with Good Manufacturing Practice (GMP), can now be created for therapeutic use ^2–4^. A global network of iPSC banks would create a strong basis for future clinical cell-based therapies ^4–9^.

The first completed clinical trial of an iPSC-derived product, assessing the safety and efficacy of CYP-001 cells (mesoangioblasts) in humans, has been reported.^10^ There are now many clinical trials in progress,^11, 12^ predominantly in the US and Japan, including administration of platelets generated from autologous iPSC cells for treatment of thrombocytopenia,^13^ sheets of iPSC-derived cardiomyocytes to treat heart disease,^14^ first-in human trials of iPSC-derived neural cells to treat subacute spinal cord injury, and dopaminergic neuron precursor cells to treat Parkinson’s disease.^15–19^ There is an increasing number of clinical trials targeting cancer using iPSC-derived immune cells. As well as direct treatments for cancer, iPSC cell lines can also be used to develop regenerative therapies aimed at repairing and supporting tissues after chemotherapy and radiation therapy.

The BMDI Cord Blood Bank (CBB) is one of three government-funded unrelated cord blood banks in Australia that together with the Sydney Cord Blood Bank and Queensland Cord Blood Bank at the Mater, in collaboration with the Australian Bone Marrow Donor Registry (ABMDR), form the AusCord Network of public cord blood banks. The CBB collects umbilical cord blood (CB) which is stored and used for bone marrow transplantation for the treatment of leukemia and other blood disorders. The CBB commenced operations in December 1996 and has more than 11,600 CB units (CBU) listed for search on the international bone marrow donor registries. The CBB is licensed by the Therapeutic Goods Administration (TGA) and is internationally accredited through the Foundation for the Accreditation of Cellular Therapy (FACT). All banked CB has been screened for safety and quality.

CB is an ideal source of starting cells for iPSC generation, with a more immature ontogeny than bone marrow and peripheral blood cells. Furthermore, these cells have not yet been exposed to genetic and environmental damage. CB cells are highly proliferative, a favorable characteristic for creating iPSC and are readily re-programmed with an overall efficiency higher than in other cell types. Only a small number of CB cells are needed for re-programming and CB iPSC have been successfully created from CB mononuclear cells (MNC) using small volumes of cryopreserved CB.^3, 20–23^

There are many aspects to consider in establishing a master bank of iPSC lines with common homozygous Human Leukocyte Antigen (HLA) haplotypes (ie, a haplobank). Best practice for the collection of human tissue samples, data management, quality control (QC), and a legal framework that is both compliant with local policy and simplifies transfer to external users must be part of, and not additional to, the core infrastructure that manages routine tasks.^9, 24^ These include the type of cells to bank, which samples to use, compliance with regulatory and ethical guidelines and clear and transparent donor consent. iPSCs are considered to be an ethically advantageous cell type, however possible issues of privacy, the immortality of cell lines as well as the involvement of commercial partners need to be considered. Rights of ownership and consent (both maternal and child), and what uses the cells will be put to are also considerations for some donors.

HLA-matching of products used for cellular therapies is an important consideration with respect to minimizing the requirement for life-long immunosuppression in order to avoid immune rejection of therapeutic cells. Banked CB cells from the Australian population, with known HLA typing, present an ideal starting point for the establishment of a bank of clinically compliant, GMP—grade iPSC lines. These lines could be used to facilitate clinical research and develop new cell-based therapies as part of a global network of iPSC banks using HLA homozygous haploidentical cells. It is envisaged that a global registry of iPSC lines of a particular haplotype be available for potential clinical use, in a manner analogous to that of the bone marrow donor registries. [www://hpscreg.eu, Table 1].

**Table 1.** A brief overview of iPSC banks worldwide derived from various cell types (modified from Huang et al^25^).

Bank name	Location	Ownership	Number of iPSC lines
Californian Institute for Regenerative Medicine	USA	Government	1556
Coriell	USA	Government	91
Fujifilm Cellular Dynamics Inc.	USA	FujiFilm	N/A
Centre for iPS Cell Research and Application	Japan	Government	22
European Bank for Induced Pluripotent Stem Cells	Europe	European Commission, private enterprises	815
Human Induced Pluripotent Stem Cell Initiative	U.K.	Medical Research Council, Wellcome Trust	835
Taiwan Human Disease iPSC Consortium	Taiwan	Government	78
RIKEN	Japan	Government	480
Korean Stem Cell Bank	Korea	Government	15
WiCell	USA	University of Wisconsin-Madison	1316

Given that there is a high level of regional variability of HLA types and that frequency of HLA haplotypes varies between populations, less common homozygous HLA haplotypes may be important for treating diseases in those from ethnic minorities within a population and should also be considered in the decision of which cell lines to create.^26^ A global haplobanking network, targeting HLA matching via banked iPSC lines worldwide could reduce redundancy to make the best use of limited resources.^2, 6–8, 15, 26–28^ An effort is being made by the International Stem Cell Banking Initiative (ISCBI) and the Global Alliance of iPSC Therapies (GAiT) to standardize Quality parameters, in order to provide safe clinically complaint GMP grade iPSC.^27,29–31^ GAiT has over 500 members from 17 countries and continues to expand. This includes Japan, England, the US, South Korea, Germany, Brazil, Spain, France, India, Singapore, and Malaysia; the membership distribution reflects where the cell therapy and industry work is being undertaken. Currently, there are no African groups as members, although some suitable haplotypes would be available through American, European, and Australian banks.

Many countries are pursuing the idea of creating HLA homozygous iPSC from banked cord blood, confirming global interest for this type of cell line.^9, 28, 32^ CB stored in the BMDI CBB searchable inventory was collected and banked with consent from CB donors for their CB to be used for unrelated bone marrow transplants, with most donors also providing consent for Quality Control or ethically approved research use if their CB is not suitable for transplant. This consent is not sufficient to allow banked CB to be used to create iPSC lines for potential therapeutic applications, and therefore requires re-consent of CB donors for this purpose. This project aims to describe the pathway and processes for establishing clinical-grade iPSC lines from banked cord blood from an ethical and governance standpoint, including the re-consent of existing donors. The critical milestones for this project were achieved through an extensive review of the literature and global presentations, discussion and a research-based consultation that included scientists and experts from various aspects of stem cell and cell therapies, including legal.

## Methods

### Literature Review and Consultation With Members of International Societies

An in-depth review of the literature around human ethics and consent, ethics and stem cells, iPSC and cell banking was undertaken, assessing, and reviewing papers, conference notes and symposiums, ethics forums and established guidelines looking at embryonic stem cells (ESC), cell line creation and the associated ethical, regulatory and governance processes. The ethics associated with consent involving open ended research and dynamic consent for genotyping and interrogation of genomics was also examined. The aim was to identify the areas of concern for researchers and medical practitioners as well as the general public. Strong attention was paid to areas such as consent, privacy, transparency and potential commercialization.

To obtain a broad perspective, a round table collaboration with members of the wider scientific community was undertaken to discuss the issues and potential roadblocks for ethics approval, consent and governance. Included at the table were stem cell scientists familiar with both iPSC and cellular therapies, lawyers specializing in areas of governance and ethics/consent (both National and International) including biobanking, medical practitioners, ethicists, cord blood bank staff, Directors/Scientific Directors of all three Australian Public cord blood banks and experts with GMP)/Good Clinical Practice (GCP) knowledge, and members of FACT. As well as identifying issues, the discussion touched upon potential community acceptance and the concerns with consent dealing with children and guardians as part of the proposal to re-consent donors to use their stored cord blood.

### Identifying Donors

Local institutional Human Research Ethics Committee (HREC) approval was sought and obtained to create iPSC from banked CB following a period of consultation with representatives of the HREC and the Victorian Clinical Genetic Services (VCGS). In principle approval was also sought and obtained from the ABMDR, as the body with contractual oversight over the public cord blood banks, and the Commonwealth government, who fund the public cord blood banking program. CB units within 10 years of cord blood donation were selected in order to increase the likelihood that consent, and any relevant follow-up health information could be obtained from the maternal donor. A caveat was included in the Donor Information Consent Form (DICF) that it was the donor’s responsibility to inform their child when they reach maturity that their cells may be used for clinical research or therapies. Due to the potential for iPSC to exist for an extended period of time, it was suggested that the child has a right to know when they came of age if their cells have been used. As this would be well past the withdrawal period, it would be as a courtesy and extremely difficult for any researcher to trace, so is the responsibility of the parents.

### HLA Haplotyping and Selection of Donors

For this study, potential donor candidates were selected based on existing low-resolution HLA data in the CBB, which was confirmed by Next Generation Sequencing (NGS) across 11 HLA loci. HLA tissue typing data from CB banked at the BMDI CBB are tested and held by the Victorian Transplantation and Immunogenetics Service (VTIS) of Australian Red Cross Lifeblood and was interrogated using a purpose-written algorithm (see Supplementary Information).

### Informed Consent and Ethics Approval for Re-consent of Donors

Donors identified as having CB with a HLA homozygous haplotype were mailed a letter containing a Donor Information and Consent Form in simple language combined with pictures (refer to Supplementary Information 1), a permission to contact slip to be filled in to allow contact and follow up with the donor and a stamped return envelope addressed to the CBB for the consent form. Following the letter and receipt of approval from the donor to be contacted, there was a phone call followed by an interview and time to ask questions before obtaining donor consent, which was then verified by the CBB staff member. A summary of the items the donors were asked to consent to is outlined in Table 2. Once contact had been established a phone interview was set up where donors could ask any questions they had about the project or what they were consenting to. If donor contact was agreed to by return of the slip, phone contact was attempted three times before ceasing follow-up attempts as we did not want to risk the annoyance of donors. Not sending back the slip was also considered a “no”. As the Australian Cord Blood Banks do not currently collect cord bloods from non-English speaking donors, a translator service was not required.

**Table 2.** Consent form articles from the DICF form. A complete copy of the DICF can be found in the supplementary material.

•	I have read or had read to me the information sheet about this project. I have had the opportunity to ask any questions and am satisfied with the answers I have received
•	I have had the chance to consider the information and to discuss any concerns with individuals who are independent of the project
•	I have been advised that the researcher will conduct this research in a manner conforming to ethical and scientific principles set out by the National Health and Medical Research Council of Australia (NHMRC)
•	Some of the data from cell lines may be made available to others in a de-identified form that protects my privacy as a quality control measure
•	I understand that I may be re-contacted in the future if information of direct importance to my health or my family’s health becomes available
•	I understand that any stem cell lines made may be stored indefinitely for clinical study use and quality control purposes
•	I understand that any iPSC lines created will be owned by the researchers and may be distributed to other laboratories in Australia and overseas for the use in clinical research
•	I understand that I can withdraw from the study without giving any reason up until the time my cells have been used to make iPSC cells and this will not affect my relationship with the BMDI Cord Blood Bank, any medical care or legal rights
•	I agree that research data gathered from the results of the project may be published, provided that I cannot be identified
•	I understand that if my sample or data could be of use for another study I will be contacted for further consent before the sample is used
•	I understand that if a stem cell line is made that it will be stored in a Stem Cell Bank and that it may be used in the development of clinical treatments
•	I understand that quality testing required to ensure that cells are viable (alive), of good quality and safe to use may be performed on the cells created from my donation
•	I understand that cell lines or discoveries made using them may be valuable but that I will not benefit financially from taking part
•	I understand that if new treatments are developed from stem cell lines, I cannot say who will get the treatment
•	I will be given copies of the Participant Information and the Consent to Participate in Research Forms

### Targeted Gene Sequencing

Targeted gene sequencing (TGS) will be performed on the stored CB sample, as an up-front test of genomic integrity, and on the final iPSC line for the master bank, as a Quality Assurance check to ensure that the ex vivo manipulation and re-programming of the cells has not caused genomic aberrations in the final iPSC product that may potentially be used for clinical studies or therapeutic use. It is proposed to probe 18 genes implicated in various conditions such as cancer or genomic instabilities that could lead to other disorders, based on published literature and advice from the Victorian Clinical Genetics Service (VCGS). Table 3 lists the genes to be interrogated. Cells will be tested both before and after reprogramming to identify changes incurred during reprogramming. Cell lines demonstrating such changes will not be progressed or used.

**Table 3.** Summary of the 18 genes to be probed using target gene sequencing and their related effects/conditions.

Gene	Encodes for	Potential clinical association with expression changes
*TP53*	Tumor suppressor gene	Heterozygous mutations cause Li Fraumeni syndrome with high risk of cancer in children and adults
*HAUS 7*	This gene encodes a subunit of the augmin complex, which regulates centrosome and mitotic spindle integrity, and is necessary for the completion of cytokinesis	Alterations in cytokinesis have been associated with various cancers
*ZFYVE16*	This protein functions as a scaffold protein in the transforming growth factor-β signaling pathway and is involved in regulation of the bone morphogenetic protein signaling pathway	Variations may be associated with metastatic gastric adenocarcinoma
*PLAA*	Phospholipase A2-activating protein (PLAA, or PLAP) is potentially important in regulating the inflammatory response through its activation of phospholipase A2	Homozygous/compound heterozygous mutations cause a progressive neurodevelopmental disorder
*MPRIP*	Targets myosin phosphatase to the actin cytoskeleton. Required for the regulation of the actin cytoskeleton by RhoA and ROCK1	Variants may affect cytoskeletal development of cells
*PIN1*	The enzyme binds to a subset of proteins and thus plays a role as a post phosphorylation control in regulating protein function. Studies have shown that the deregulation of Pin1 may play a pivotal role in various diseases	The up-regulation of Pin1 is implicated in certain cancers, and variants in Pin1 are associated with Alzheimer’s disease
*CYP4F2*	The cytochrome P450 proteins are monooxygenases which catalyse many reactions involved in drug metabolism and synthesis of cholesterol, steroids, fatty acids, and other lipids	Variants are associated with hypertension and ischemic stroke
*SLC23A2*	This gene encodes one of the two required transporters and the encoded protein accounts for tissue-specific uptake of vitamin C	Polymorphisms associated with glaucoma and gastric cancer as well as altering levels of vitamin C adsorption
*TAF 1*	This gene encodes the largest subunit of TFIID. This subunit binds to core promoter sequences encompassing the transcription start site. It also binds to activators and other transcriptional regulators, and these interactions affect the rate of transcription initiation	Hemizygous mutations cause intellectual disability
*CHUK*	A protein kinase that is part of the IκB kinase complex that plays an important role in regulating the NF-κB transcription factor	Homozygous/compound heterozygous mutations cause Cocoon syndrome
*EEF1A2*	An isoform of the alpha subunit of the elongation factor-1 complex, which is responsible for the enzymatic delivery of aminoacyl tRNAs to the ribosome	Heterozygous mutations cause intellectual disability with or without epileptic encephalopathy
*MAP2*	MAP2 serves to stabilize microtubules growth	Variations may be associated with neuronal development disorders
*BSN*	A scaffolding protein involved in organizing the presynaptic cytoskeleton	Uncertain, may play a role in neuronal development disorders
*MED12L*	Mediator of RNA polymerase II transcription, subunit 12 homolog (X chromosome)	Uncertain, markers are found in neural/mental syndromes, prostate cancer, leiomyomas and breast fibroepithelial tumors
*SPRY1*	Sprouty RTK signaling antagonist 1	Various cancers: prostate, breast ovarian, Liver Uncertain, may play a role as a tumor suppressor gene
*ATP10B*	ATP binding, magnesium ion binding phospholipid translocating ATPase	Various cancers: head and neck, stomach, colorectal Variants associated with colorectal cancer
*AHI1*	Aa protein coding gene that is known for the critical role it plays in brain development	Homozygous/compound heterozygous mutations cause Joubert syndrome, associated with intellectual disability
*MYH7*	Myosin heavy chain beta (MHC-β) isoform (slow twitch) expressed primarily in the heart, but also in skeletal muscles (type I fibers)	Heterozygous mutations cause cardiomyopathy and/or skeletal muscle disorders
*AGBL1*	A glutamate decarboxylase that catalyses the deglutamylation of polyglutamylated proteins	Heterozygous mutations cause corneal dystrophy

## Results

### HLA Haplotyping

**Figure 1.**
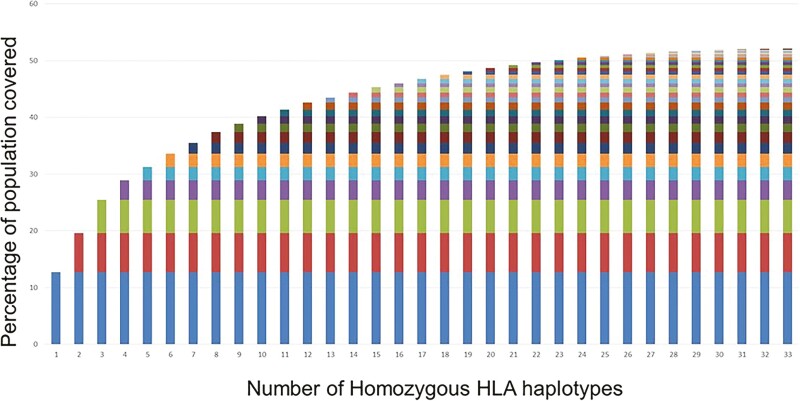
Cumulative coverage of the local population by the homozygous haplotypes identified in the BMDI Cord Blood Bank. Each color represents a unique haplotype. There is a minimal increase in coverage after the first 23 haplotypes identified.

From a total of 13,679 CBU records interrogated, at least 143 CBU with homozygous haplotypes at the 2-digit level of HLA-A, B, and DRB1 were identified, with 23 CBU with unique homozygous haplotypes. Each homozygous haplotype could then be matched to either itself or one of six heterozygous HLA haplotypes. When these haplotypes were used to probe the existing units in the BMDI CBB database, it was found that 6,768 units had one haplotype match to the homozygous HLA haplotypes; at least 50% of the local population could be covered by iPSC lines derived from the 33 unique HLA homozygous haplotypes identified (Figure 1). As with other studies of this type, the data are representative of the population serviced by the BMDI CBB and may not reflect the larger Australian population. The most common homozygous HLA haplotypes are similar to those identified in the UK population by Taylor et al^7^ and which align with that observed across the Australian public cord blood banking network (AusCord) (Table 4).^33^ Eighteen CBU with homozygous haplotypes were identified as having been collected in the last 10 years, with suitable maternal and donor family and medical history, and acceptable long-term donor follow-up obtained. Next generation sequencing was performed on the selected 18 CBU to confirm the low-resolution HLA haplotype identification. All samples were confirmed to be homozygous at the HLA-A, -B, -C, DRB1, DRB3, DQA1, and DQB1 loci with 3 CBU maintaining homozygosity at the DPB1 loci. The most commonly identified HLA homozygous haplotype was HLA-A*01:01; B*08:01; C*07:01; DRB1*03:01; DRB3*01:01; DQA1*05:01; DQB1*02:01.

**Table 4.** Comparison of the top 7 HLA homozygous haplotypes identified in the British population by Taylor et al^7^ and those identified in the BMDI CBB, Melbourne Australia, and the AusCord network.^33^

United Kingdom top 7 HLA homozygous haplotypes	BMDI CBB top 7 HLA homozygous haplotypes	Auscord top 7 HLA homozygous haplotypes
1, 8, 3	1, 8, 3	1, 8, 3
2, 44, 4	2, 44, 4	2, 44, 4
3, 7, 15	3, 7, 15	3, 7, 15
2, 7, 15	2, 7, 15	2, 7, 15
2, 44, 7	2, 51, 11	29, 44, 7
2, 62, 4	2, 8, 3	1, 57, 7
1, 57, 7	1, 57, 7^(^^1^^)^	2, 15, 4

There were other haplotypes at this frequency. AusCord data adapted from Klamer et al.^34^

### Donor Response

Donor re-consent DICF and contact letters were mailed to 18 women; 8 immediate responses were received granting permission to contact (44%), with 4 returned as address unknown (22%). At 8 weeks post-mail-out, 6 donors have completed the full re-consent process (33%), including our top choice for creating the first GMP-grade iPSC line. As this is an ongoing open ended consent process, these women were considered a first pass endpoint for this study.

A positive first contact response was returned by more than 50% of donors within the first 6 weeks, with 6 full consent forms signed and remaining contact interviews in progress. The primary response from donors has been an interest in the project, with some donors enquiring about potential uses for the cells. All respondents so far have been comfortable with the science and testing required of their cells and have had few questions. The most commonly asked questions were: When were the cells likely to be used? Were there any current projects and what areas of research were they in? How would potential commercial partners be involved? Most women stated that the PICF was easy to follow in terms of what an iPSC was. Several women asked whether they had to sign the consent form as well as the permission to contact slip, suggesting that the introductory letter could be clearer. Three donors signed the full consent and posted it back, without any verbal discussion with the CBB. These donors were contacted by the CBB and given the opportunity to ask any questions and to confirm that they understood what had been consented to. Contact with the donors through this process has also allowed additional long-term follow up to the original CB donation.

## Discussion

We have shown that interrogating the CBB database for suitable cord blood units (CBU), coupled with a clear and transparent re-consent process, is viable when seeking permission for potentially sensitive techniques, such as genomic testing and creation of iPSC. The BMDI CBB extended donor follow-up period allows high-level traceability of existing donors for re-consent to use their CB for other purposes. Lack of response or follow-up to contact calls appeared due to lifestyle factors rather than disagreement with the requested use for their banked CB. Working with local and International collaborators such as GAiT and ISCBI has helped lead to development of ethical and regulatory processes in order to improve donor understanding and protections which can lead to an increased willingness to participate in research studies, avoiding unintended restrictions on downstream use due to poorly worded or unclear consent taking into account local guidelines and conditions.^9^

To create iPSC lines from CBU identified as having a homozygous HLA haplotype, it is necessary to re-consent the donors identified. Some of the questions raised by the HREC was who would be using the iPSC cell lines to derive end-product cells for therapeutic use, and whether there would be questions from the donor and full disclosure regarding potential industry partnership and commercialization. To ensure transparency and informed consent there needs to be allowance for future research and technologies that are developing quickly and were not on the radar at the beginning of this study. In this case, guidance is provided via international and national guidelines such as those of the ISSCR and the National Health and Medical Research Council (Aus.) (NHMRC) which have been crafted in consultation with all sectors of the scientific community. As all future research will still have to receive ethics approval, guidelines will change to reflect community opinion and we have included the right to re-contact the donor which will enable us to provide a more informed consent on potentially contentious research.

The decision was made to include consent for open-ended research in the DICF. The iPSC and their donor material will be stored for a long time, and it is not always possible to maintain contact with donors over what may be many years. As these cells are not a single research project as such, but a resource for other projects, informed consent for each individual research project is difficult and impractical. It has been observed that many in the general public, and patient participants, prefer general consent, but that many desire to have some form of ongoing control, such as a tiered or re-consent approach, while developing a good relationship between communities improved acceptance for providing open-ended consent to biobank research.^35–37^ It is recommended to avoid inclusion of disease-associated restrictions which can limit downstream use of iPSCs, particularly as the understanding of the interconnection between multiple pathologies progresses.^9^

A thorough investigation of the literature in this field revealed that many donors are willing to provide samples and information for research into the understanding and treatment of diseases but may be reluctant to provide tissue and personal information for research into areas that may be contentious or can be seen as a profit-making exercise with no benefit to the donor, even though the CBB may only receive recovery costs. After high-profile cases such as the He-La cells controversy, donors want more transparency regarding use of their donations.^2, 38, 39^ To ensure donor acceptance and comfort with the use of their tissues, contentious areas of research, such as cloning and creation of gametes, were mentioned and specifically excluded from the consent, and donors assured that their cells would not be used for these areas of research.

The consent forms used to collect and store CB throughout the past 25 years of operation of the BMDI CBB have obtained consent from donors to use their CB for bone marrow transplantation, quality control purposes, or for research approved by a National HREC. To date, consent has not been sought from donors for the non-homologous (ie, non-hematopoietic stem cell transplant) use of their CB for therapeutic purposes, or for applications such as the creation of iPSCs. Consent has also not specifically been obtained for provision of their CB to a third-party commercial manufacturing organization (CMO); it is likely that scale-up manufacturing of CB-derived cellular therapies will require collaboration with CMOs.^38, 40^

While the desire to use TGS as part of the Quality Assurance and Quality Control for the iPSC cell lines was understood by the HREC and others, issues of privacy and incidental information disclosure were raised, including the “right to know” by extended family members of the donor if any abnormality was identified that may affect them. In consultation with the HREC, the decision was reached to ensure that the families would be referred to a genetic counseling service and re-testing undertaken to exclude for any false positive results. Clinically relevant information is defined as something that may have a direct health impact on the donor and their family’s health. Findings should have clinical utility and validity to inform the donor rather than something that is not affecting them, and they are being offered further genetic testing to confirm validity.

The rights to privacy of this information was very strongly regarded and only a de-identified summary of part of the genetic information would be provided to third party users. All information, including any personal information is strictly protected by numerous safeguards, as per The Victorian Parliament’s Human Tissue Act (Version 45), Essentially Yours: The Protection of Human Genetic Information in Australia (ALRC Report 96), the National Health and Medical Research Council (Aus.) (NHMRC), the Foundation for the Accreditation of Cellular Therapy (FACT) and Therapeutic Goods Administration (TGA) regulatory standards.^41–44^

Recent studies have demonstrated that the majority of participants who sign consent forms do not really understand the concepts of the projects outlined on the forms.^39,45–48^ In the case of stem cell research, there is limited understanding about the concepts of “stem cells”, “immortality”, “pluripotency”, and “storage” in these contexts. Genomic Testing and the amount of information it could provide is also not easily understood. While it has been suggested that it is not necessary that donors understand all the details of a project, sufficient understanding of the principles and protections included in the consent are required for trust. ^47,49^ This also highlights the possible need for repeat contact with the donor to ensure both understanding and an establishment of trust between the CBB and donor. It has been suggested by others that additional decision aids such as video may also be helpful if needed.^34,39,45^

The HLA-matching of products used for cellular therapies is an important consideration, minimizing the requirement for life-long immunosuppression in order to avoid immune rejection of the graft. This is an important aspect of identifying donors, determining which haplotypes to focus on and introduces the concept of “super donors”—HLA homozygous donors whose cells could cover a wide section of the population based on the genotypic frequency of the haplotype and its related heterozygous HLA matches. The haplobanking concept is that iPSCs derived from donors who are homozygous for a particular HLA haplotype will match any patient that carries that haplotype. If the haplotype is common in the population, many patients will benefit from this selected donor.

Reviews by Taylor et al calculated the optimal combination of homozygous HLA types that would match a majority of the UK population. They identified the most useful homozygous HLA haplotypes present among 17 million HLA-typed volunteer stem cell donors registered on Bone Marrow Donors Worldwide (BMDW) that could provide a match for 93% of the UK population. It is reasonable to assume that the figures for HLA antigen matching in Australia will be similar those in the UK as both countries share a similar majority population demographic, although different minority groupings. Indeed, the top 4 homozygous haplotypes identified by our study are identical to the top 4 identified UK haplotypes.^50^ Similar modeling in places such as Korea Japan, the US^28, 51^ and other countries such as China, Brazil India and Russia are also identifying the suitable HLA homozygous haplotypes for their populations. The heterogeneity and size of the populations in different parts of the world means that the best HLA homozygous haplotypes for maximum coverage vary from country to country. This low overlap between countries significantly increases the number of HLA homozygous haplotypes required worldwide, further supporting the need for a global network of iPSC banks working under globally acknowledged QA and QC standards as strong basis for future clinical cell- based therapies.

## Summary/Conclusions

Creation of clinically compliant iPSC lines significantly extends the purpose of altruistically donated cord blood for the treatment of patients, through the potential provision of regenerative therapies. Using small volumes of banked CB mononuclear cells (MNC), it is planned to create a master bank of GMP-grade HLA homozygous iPSC from CBU whose HLA haplotype has been verified by NGS sequencing. We have described a clearly defined ethical pathway for transparent and informed re-consent of CB donors, that reflects the current guidelines of National and International regulatory bodies as well as collaborative bodies such as the European Bank for induced Pluripotent Stem Cells (EBiSC),^9^ ISCBI, GAiT, the International Society of Stem Cell Research (ISSCR), and the International Society of Cellular Therapies (ISCT).

While the CBB will soon implement an upfront expanded consent for prospective CB collection and banking, we have demonstrated that donors of CB already in the bank are contactable and are willing to re-consent for their CB to be used for other clinical and research applications, such as iPSC creation. The creation of clinically compliant iPSC cell lines will provide an important tool for preclinical research for a range of conditions, including cancer, neurological disorders, diabetes, and cardiac disease, ultimately aiming to translate into novel therapies. Establishment of a CB-derived iPSC master bank will create a value-added service for the CBB, enhancing the existing CB supply for clinical use and increasing the number of people who can benefit from a CB donation.

## Supplementary Material

szac060_suppl_Supplementary_Data

szac060_suppl_Supplementary_Information

## Data Availability

The data that support the findings of this study are available from the corresponding author upon reasonable request.
