# Supplementary material for: Identification and Re-consent of Existing Cord Blood Donors for Creation of Induced Pluripotent Stem Cell Lines for Potential Clinical Applications
Source: Stem Cells Transl Med. 2022 Sep 8;11(10):1052–60. doi: 10.1093/stcltm/szac060 (PMC9585951; doi:10.1093/stcltm/szac060)
Supplement: szac060_suppl_Supplementary_Data [file szac060_suppl_supplementary_data.doc]

Supplementary Information: Copy of Donor Information Consent Form provided to CB donors.

Short Name of Project: Creating new stem cell lines for clinical research projects.

Full Name of Project: Creating human induced pluripotent stem cell (iPSC) lines derived from stored umbilical cord blood units as a resource to facilitate clinical research

Thank you for taking the time to read this **Donor Information Statement and Consent Form**. We would like to invite you to take part in a research project that is explained in this form.

This form is 8 pages long. Please make sure you have all the pages.

**What is an Information Statement and Consent Form?**

An Information and Consent Form tells you about the research project. It explains exactly what the research project will involve. This information is to help you decide whether or not you would like to take part in the research. Please read it carefully.

Before you decide if you want to take part or not, you can ask us any questions you have about the project. You may want to talk about the project with your family, friends or health care worker.

**Taking part in the research project is up to you**

It is your choice whether or not you take part in the research project. You do not have to agree if you do not want to.

**Signing the form**

If you would like to take part in the research, please sign the consent form at the end of this document. By signing the form you are telling us that you:

- understand what you have read
- have had a chance to ask questions and received satisfactory answers
- consent to taking part in the project.

We will give you a copy of this form to keep.

1. **What is the research project about?**

Induced “pluripotent” stem cells (iPSC) are cells that can be converted into all different kinds of cell types, such as muscle, nerve, or heart cells. These iPSC can be made in the laboratory from donated samples, such as skin, blood or hair, and can be kept alive and stored long-term in a stem cell bank. When needed, these iPSC can be thawed and used to make any cell type, such as nerve cells and heart cells. These new cells can then be used in clinical research studies to develop new cellular therapies to treat a range of diseases. Trials are already underway to use these types of cells to treat Parkinson’s disease and a type of blindness.


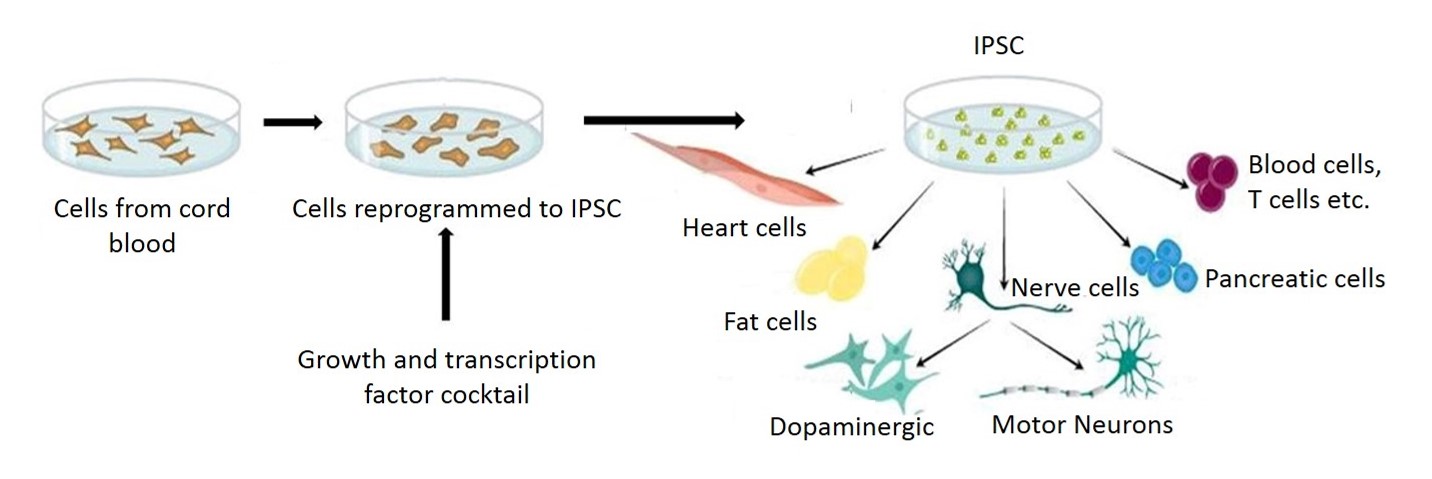


***How iPSC can be used to make new cells****. (adapted from Sharma R Dent J (Basel)*

*. 2016 Jun 6;4(2):19)*

Cord blood is a rich source of immature cells that are able to grow fast and have not yet been exposed to genetic or environmental damage. This makes the cord blood stored within a cord blood bank an excellent source of cells for making iPSC. The small amount of cord blood we would need to use (about 1 millilitre) means that the majority of the banked donation will still be available for bone marrow transplant if it is needed. Human iPSC can be grown for long periods in the laboratory and can be stored indefinitely which allows us to produce large numbers of cells that have the ability to be turned into other types of cells. By keeping an iPSC cell line frozen and looking after it carefully, it can survive indefinitely.

We would like to make iPSC from a small sample of previously donated cord blood that is held in storage at the BMDI Cord Blood Bank. If we are able to grow stem cells from these samples, they will form a group of many cells called an iPSC cell line.


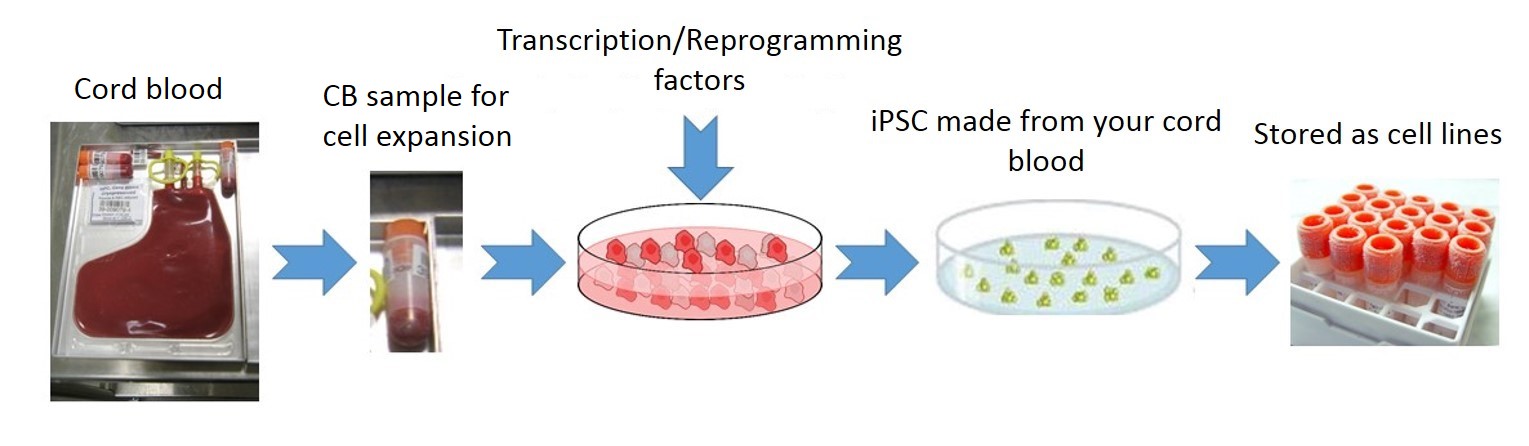


***How your cells from frozen cord blood will be used to establish an iPSC cell line***

**Release of iPSC cell lines to other researchers:**

The aim of this project is to make clinical grade iPSC cell lines. Other researchers can then take these iPSC and turn them into any cell type they need in order to develop new methods of curing or correcting diseases. Examples of the types of cells that could ultimately be made for clinical use are nerve cells, heart cells, muscle cells, retinal (eye) cells and insulin-producing cells. These new cells will be tested in the laboratory and in some animal models to ensure the cells do what they are designed to do. The cells may then be used to treat patients.

Each request to use our iPSC cell lines will undergo scientific and ethical review by a registered human research ethics committee before the cell lines are released. Your cells **will not** be used for any of the following: creation of gametes (eggs and or sperm), reproductive cloning (i.e. cloning of people) or any research not approved by the ethics committees.


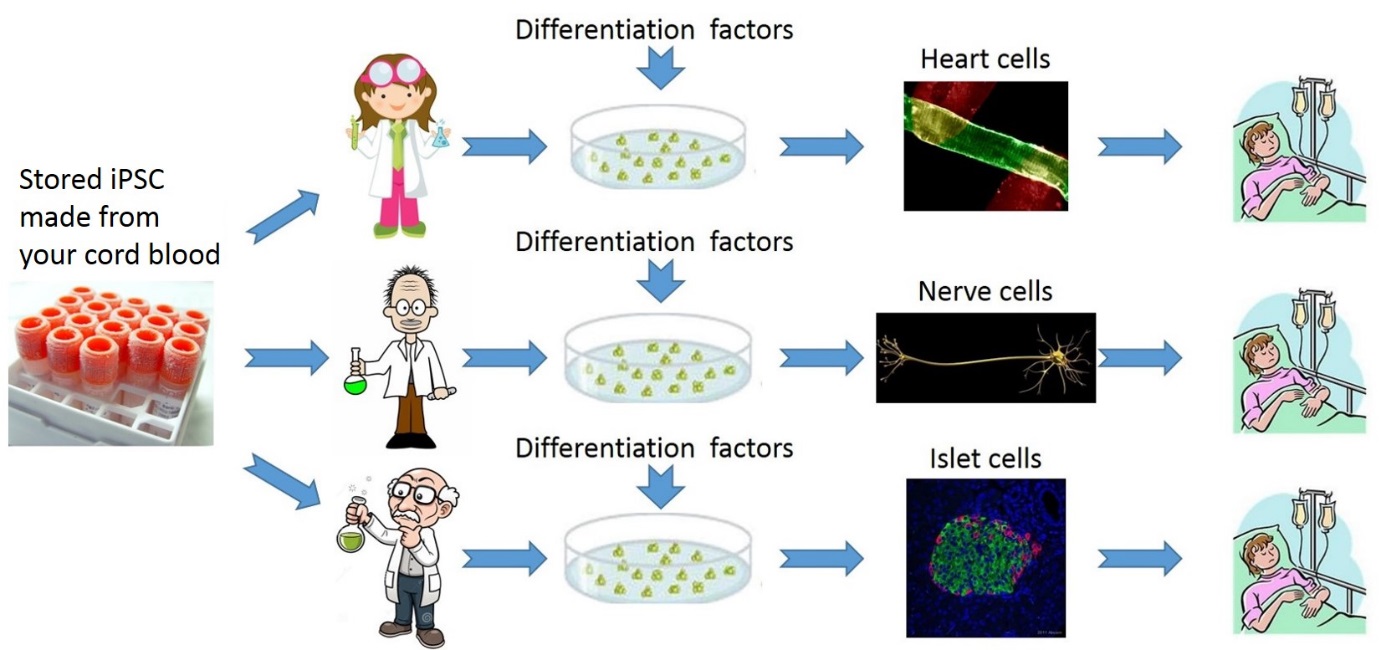


***Other Researchers and Clinicians will use the banked iPSC to create other cell types such as heart cells and pancreatic islets. These cells will then be used for clinical trials and research.***

1. **Who is running the project?**

This project is being run by the Director of the BMDI Cord Blood Bank, which is run in partnership by the Murdoch Children’s Research Institute, The Fight Cancer Foundation and the Royal Children’s hospital. This project has been approved by an ethics committee and will be governed by the principles of the NHMRC “National Statement on Ethical Conduct in Human Research” covering the use of human tissues and clinical studies.

1. **Why am I being asked to take part?**

You are being asked to participate in this project because you have already donated cord blood to the BMDI Cord Blood Bank. Your banked cord blood meets all relevant international donor selection and health screening standards. Importantly your tissue type may be useful for a significant number of patients for several different types of clinical research and possible therapies.

1. **What does this involve for me?**

We are asking permission to use a small amount of the cord blood sample (approximately 1 ml) that you have already donated. We do not require any more blood from you. We may ask you some questions about your medical history that will be similar to the follow up questions you were asked after your original donation.

We will then test your cord blood to see if it is suitable to create the iPSC cells from. We have specific requirements for the cord blood we will choose to make iPSC from and some of these are different to those required for standard cord blood donation. If we do not choose to use your blood, it will still be suitable for potential bone marrow transplant and will remain in the Cord Blood Bank. We will let you know if we choose to use your blood to make iPSC from.

As part of this process we will use a genetic test known as whole genome sequencing (WGS). This means we will take some of the DNA from your cord blood sample to run a screen on your genes. Your genes contain the instructions for growth and development of the body and each person’s genome contains many genetic differences. Most of these are harmless and have no impact on the gene’s function.

We will not be looking at all your genes, but at a set of 18 genes. Some of these genes are involved in different aspects of cell development and function, while changes in others may be related to different types of cancer, heart and nerve disorders. Each set of cells will be tested twice- once when the cord blood unit is selected and again after the iPSC have been created and expanded in culture. It is possible that ***if*** we see any changes in the genetic testing that these changes could be caused by how we grow the cells in the laboratory. We therefore screen the cells twice to monitor any potential changes.

There is a ***very small*** risk that we will find some information that is clinically relevant to you and your children. If this happens we will pass on this information to you and refer you to the Victorian Clinical Genetic Services who will assist you with any questions and arrange re-testing for your child, to confirm whether the significant finding was real or caused by culture conditions. Counselling will also be made available if necessary.

A summary of part of this genetic information may be included in a certificate of analysis for the iPSC and given to collaborators or users of the iPSC cell line but this information will be de-identified and will not be able to be linked to you.

1. **Can I withdraw from the project?**

You can stop taking part in the project prior to the cells being created and banked. You just need to tell us so. You do not need to tell us the reason why. We are able to honour such requests on the following basis:

• You may withdraw your consent to the use of all of your original donation until the point at which these have been used to create iPSC cells.

• You may withdraw your consent to be contacted by us about this study in the future, unless information critically related to the health of yourself, your family or the public is found.

• Once the iPSC cells are made they will not be destroyed. If the iPSC have been distributed to others, they will not be retrievable and may continue to be used in the development of new treatments and will potentially benefit a broad range of projects in the future. Withdrawing the cells at this stage would mean the loss of experimental data and a significant waste of time, costs and resources

1. **What are the possible benefits for me and other people in the future?**

The small sample of cord blood required to make the iPSC cell line means that your banked cord blood unit will still be available for bone marrow transplant, but the number of people who could benefit from your donation may increase.

Participation in this study will not benefit you or your family directly. It will take a long time for research to progress and to yield safe, effective treatments based on iPSC cells obtained in this study. Your donation of cells will support medical research to better understand various diseases and develop better treatments, which may help you or others in the future.

There will be no financial compensation for participating in this project. You will not have any rights to the created cell lines or any further work or products generated downstream from this project. With appropriate ethics approvals, the cell lines will be available to national and international clinical research groups, or contract manufacturing organisations, and you will have no say on who can or cannot use the cells.

1. **What are the possible risks, side-effects, discomforts and/or inconveniences?**

There are no physical risks or side effects involved for you as the cord blood sample has already been collected.

We are aware that there may be privacy concerns in any research. Some of the results of this study may be published in the medical literature. We will not publish WGS data and we will not release this data to other researchers apart from the summary information required to provide a certificate of analysis for the cells. When we publish results, we do not use names or personally-identifiable information. It would be very difficult to identify any individual based on such published data. Every effort will be exerted to maintain data security and privacy.

1. **What will be done to make sure my information is confidential?**

There are strict legal requirements on donor confidentiality. Your name, birth date, and other personally-identifying information will be removed from your data and samples. They will be linked to your sample only by a code number. The code key for the samples will be stored in password-protected database under control of the BMDI Cord Blood Bank investigators. Medical information, samples, and cells that are shared with others will be coded and will not include identifying information (name, address, telephone number, or personal identification number). Only the original Cord Blood Bank will be able to trace your samples and information to you.

All information we receive is stored on a password protected electronic data base and server in the BMDI Cord Blood Bank (BMDI CBB) and can only be accessed by authorised personnel. The cord blood records at the BMDI CBB are stored in a secure facility in accordance with both the Therapeutic Goods Administration and current good manufacturing process (GMP) licence requirements. Any new information collected as a result of re-consenting you or as part of this proposal will be added to these existing files. These records must be stored indefinitely.

1. **Will I be re-contacted or receive any feedback on the programme?**

We may need to contact you in the future. This can be achieved while keeping your confidentiality using the de-identifying and coding system described above. Please remember to update the Cord Blood Bank with your contact information if it changes. Otherwise, they may not be able to find you.

You may be re-contacted for one of the following reasons:

We will contact you to let you know if we have made iPSC lines from your sample. At this time we do not plan to re-consent your child about the use of their cells when they reach 18. It is your responsibility to inform your child that their cells are being used for research when they reach maturity

We may contact you to request updates on your health. If we do you are under no obligation to provide additional information.

We may discover that the iPSC cells made from your donation are appropriate for research that is not covered by this consent form, and want to obtain your permission to use them in this new way. It might include some research conducted for new purposes or involve new techniques that we cannot at this time foresee.

1. **Who should I contact for more information?**

If you would like more information about the project, please contact: *Contact details provided*

| You can contact the Director of Research Ethics & Governance at The Royal Children’s Hospital Melbourne if you:   - have any concerns or complaints about the project - are worried about your rights as a research participant - would like to speak to someone independent of the project.   The Director can be contacted by telephone on XXXXX |
| --- |

**CONSENT FORM**

| **HREC Project Number:** | XXXXX | | |  |
| --- | --- | --- | --- | --- |
| **Short Name of Project:** | Creating new stem cell lines for clinical research projects. | | | |
| **Version Number:** | #3 | **Version Date:** | 16/04/2019 | |

1. I have read or had read to me the information sheet about this project. I have had the opportunity to ask any questions and am satisfied with the answers I have received.
2. I have had the chance to consider the information and to discuss any concerns with individuals who are independent of the project.
3. I have been advised that the researcher will conduct this research in a manner conforming to ethical and scientific principles set out by the National Health and Medical Research Council of Australia (NHMRC).
4. Some of the data from cell lines may be made available to others in a de-identified form that protects my privacy as a quality control measure.
5. I understand that I may be re-contacted in the future if information of direct importance to my health or my family’s health becomes available
6. I understand that any stem cell lines made may be stored indefinitely for clinical study use and quality control purposes.
7. I understand that any iPSC lines created will be owned by the researchers and may be distributed to other laboratories in Australia and overseas for the use in clinical research.
8. I understand that I can withdraw from the study without giving any reason up until the time my cells have been used to make iPSC cells and this will not affect my relationship with the BMDI Cord Blood Bank, any medical care or legal rights.
9. I agree that research data gathered from the results of the project may be published, provided that I cannot be identified.
10. I understand that if my sample or data could be of use for another study I will be contacted for further consent before the sample is used.
11. I understand that if a stem cell line is made that it will be stored in a Stem Cell Bank and that it may be used in the development of clinical treatments.
12. I understand that quality testing required to ensure that cells are viable (alive), of good quality and safe to use may be performed on the cells created from my donation
13. I understand that cell lines or discoveries made using them may be valuable but that I will not benefit financially from taking part.
14. I understand that if new treatments are developed from stem cell lines, I cannot say who will get the treatment.
15. I will be given copies of the Participant Information and the Consent to Participate in Research Forms.

| I do | I do not | Agree to take part in the above study and understand that I am under no obligation to do so. |
| --- | --- | --- |

| Participant Name |  | Participant Signature |  | Date |
| --- | --- | --- | --- | --- |

| Name of Witness to Participant’s Signature |  | Witness Signature |  | Date |
| --- | --- | --- | --- | --- |

**Declaration by researcher:** I have explained the project to the participant who has signed above. I believe that they understand the purpose, extent and possible risks of their involvement in this project.

| Research Team Member Name |  | Research Team Member Signature |  | Date |
| --- | --- | --- | --- | --- |

Note: All parties signing the Consent Form must date their own signature.
