# Supplementary material for: Identification and Re-consent of Existing Cord Blood Donors for Creation of Induced Pluripotent Stem Cell Lines for Potential Clinical Applications
Source: Stem Cells Transl Med. 2022 Sep 8;11(10):1052–60. doi: 10.1093/stcltm/szac060 (PMC9585951; doi:10.1093/stcltm/szac060)
Supplement: szac060_suppl_Supplementary_Information [file szac060_suppl_supplementary_information.docx]

***HLA Interrogation:*** HLA tissue typing data from the BMDI Cord Blood Bank was interrogated using a purpose-written algorithm. To begin the analysis, all the unique haplotypes that were homozygous for the three loci (A, B, DRB1) in the sample (at 1-field/2-digit resolution) were ascertained for at least one individual. These were ordered by the number of homozygous samples that carry that haplotype. This was called the homozygous haplotypes list. Starting with the most common haplotype in this list the number of individuals in the whole sample who matched that haplotype for at least one allele at each of the three loci (A, B, DRB1) was counted. This count was stored in a table (indexed by the haplotype). All of the matching individuals from the sample were then removed (as a match had already been found for them), and the next most common haplotype in the homozygous haplotypes list was considered. The number of individuals in the remaining sample who matched that (next most common) haplotype for at least one allele at each of the three loci was counted, the count again stored, and the matching individuals removed, before moving on to consider the next most common haplotype in the homozygous haplotypes list. This process was continued until either all samples have found a match, or all of the haplotypes in the homozygous haplotypes list had been tested. It is noted that this process is possibly not optimal (for example, for equally frequent haplotypes in the homozygous haplotypes list the first listed haplotype was taken to test, and furthermore it is possible that a haplotype further down the list may match more samples than one further up the list) but we contend that any departure from optimality would be minor and we certainly found a lower bound for performance in our sample. We calculated probabilities as simple frequencies of the matches for the whole sample.
